# Examining the association of life course neurocognitive ability with real-world functioning in schizophrenia-spectrum disorders

**DOI:** 10.1016/j.scog.2022.100254

**Published:** 2022-04-26

**Authors:** Sylvia Romanowska, Michael W. Best, Christopher R. Bowie, Colin A. Depp, Thomas L. Patterson, David L. Penn, Amy E. Pinkham, Philip D. Harvey

**Affiliations:** a Department of Psychological Science, University of Toronto Scarborough, Toronto, ON, Canada; b Department of Psychology, Queen's University, Kingston, ON, Canada; c Department of Psychiatry, UCSD Medical Center, La Jolla, CA, United States; d San Diego VA Healthcare System, San Diego, CA, United States; e Department of Psychology, University of North Carolina at Chapel Hill, Chapel Hill, NC, United States; f School of Behavioural and Health Sciences, Australian Catholic University, Melbourne, VIC, Australia; g Department of Psychology, University of Texas at Dallas, Dallas, TX, United States; h Department of Psychiatry, UT Southwestern Medical Center, Dallas, TX, United States; i University of Miami Miller School of Medicine, Miami VA Healthcare System, United States

**Keywords:** Psychosis, Schizophrenia, Neurocognition, Functioning, Cluster analysis

## Abstract

There is considerable variability in neurocognitive functioning within schizophrenia-spectrum disorders, and neurocognitive performance ranges from severe global impairment to normative performance. Few investigations of neurocognitive clusters have considered the degree to which deterioration relative to premorbid neurocognitive abilities is related to key illness characteristics. Moreover, while neurocognition and community functioning are strongly related, understanding of the sources of variability in the association between these two domains is also limited; it is unknown what proportion of participants would over-perform or under-perform the level of functioning expected based on current neurocognitive performance vs. lifelong attainment. This study examined data from 954 outpatients with schizophrenia-spectrum disorders across three previous studies. Neurocognition, community functioning, and symptoms were assessed. Neurocognitive subgroups were created based on current neurocognition, estimated premorbid IQ, and degree of deterioration from premorbid using z-score cut-offs; functional subgroups were created with cluster analysis based on the Specific Level of Functioning Scale and current neurocognition. The sample was neurocognitively heterogeneous; 65% displayed current neurocognitive impairment and 84% experienced some level of deterioration. Thirty percent of our sample was relatively higher functioning despite significant neurocognitive impairment. Individuals with better community functioning, regardless of neurocognitive performance, had lower symptom severity compared to those with worse functioning. These results highlight the variability in neurocognition and its role in functioning. Understanding individual differences in neurocognitive and functional profiles and the interaction between prior and current cognitive functioning can guide individualized treatment and selection of participants for clinical treatment studies.

Neurocognitive impairments are a core feature of schizophrenia (Kahn and Keefe, 2013; Fioravanti et al., 2012; Schaefer et al., 2013) across the lifespan. Impairment in multiple neurocognitive domains is commonly reported in young people at clinical high risk (CHR) for psychosis (Mesholam-Gately et al., 2009), at the first episode of psychosis (Fu et al., 2017), and throughout later phases of illness (Meier et al., 2014). However, there is considerable variability in neurocognitive functioning. While 70–84% of individuals with schizophrenia experience impairment of at least 1 standard deviation (SD) below normative standards in multiple neurocognitive domains (Nuechterlein et al., 2004; Palmer et al., 2009), 20–25% of individuals with schizophrenia demonstrate average or above-average performance on composite measures of neurocognitive function (Keefe and Fenton, 2007; Kremen et al., 2000; Palmer et al., 1997; Weickert et al., 2000). This subgroup has better clinical and functional outcomes than those with impaired neurocognition (Helldin et al., 2020; Kenney et al., 2015; Moradi et al., 2018), congruent with evidence that neurocognition is a strong predictor of community functioning for individuals with schizophrenia (Bowie and Harvey, 2006; Fervaha et al., 2014). However, the association of neurocognitive variability with functioning remains unclear.

Neurocognitive impairments in schizophrenia fit into several clusters, ranging from severe generalized impairment to intact performance. Distinct neurocognitive profiles are identifiable by the first episode (Reser et al., 2015; Uren et al., 2017) and constitute 2–5 clusters representing un-impaired, globally impaired, and mixed subgroups (Heinrichs and Awad, 1993; Horan and Goldstein, 2003; Joyce et al., 2005; Ohi et al., 2017). Generally, participants in the most globally impaired clusters have lower educational attainment, more severe psychotic symptoms, and poorer community functioning compared to those who are neuropsychologically normal or have mixed profiles of impairment (Lewandowski et al., 2014, Lewandowski et al., 2018).

Neurocognitive heterogeneity remains consistent regardless of the number of episodes of psychosis experienced (Sauvé et al., 2018). Cluster membership may also be associated with differential prognosis, with neuropsychologically normal individuals showing the best lifetime response to treatment on symptoms and functioning (Gilbert et al., 2014). Similar neurocognitive subgroupings are observable in unaffected siblings of individuals with schizophrenia (Hoti et al., 2004; Quee et al., 2014), in young people at CHR (Velthorst et al., 2019), and in children of individuals with schizophrenia (Peredo et al., 2018).

Although 20–25% of individuals with schizophrenia demonstrate average neurocognitive abilities, this does not account for whether participants have deteriorated relative to their premorbid abilities. Prospective population-based samples often manifest significant neurocognitive deterioration between childhood and psychosis onset (Kremen et al., 2010; Meier et al., 2014). Notably, Keefe et al. (2005) found that up to 98% of individuals with schizophrenia had poorer neurocognitive functioning than would be expected based on their estimated premorbid IQ and parental education.

Cluster analyses have also been used to compare current neurocognitive functioning compared with premorbid neurocognitive abilities. Individuals with “preserved” neurocognition, defined as both average premorbid and current neurocognition, have intact performance. Another group of individuals manifest “deteriorated” neurocognition, defined as more impaired current neurocognition relative to premorbid estimates. A final group, “compromised” neurocognition, is defined by both low premorbid and current neurocognitive abilities (Weickert et al., 2000). These clusters have been replicated in independent samples (Badcock et al., 2005; Wells et al., 2015), with differences on measures of functional competence (Ammari et al., 2014), community functioning, and symptom severity (Wells et al., 2015). For example, participants in compromised subgroups have more severe negative symptoms, are less likely to be employed, and have fewer friends than preserved subgroups (Leeson et al., 2011; Wells et al., 2015).

The association between neurocognitive abilities and community functioning is well established (Helldin et al., 2020; Lepage et al., 2014) and differences in functioning have been noted between clusters. Globally impaired or compromised subgroups typically display the most impaired community functioning (Uren et al., 2017). However, when examined as a continuous variable, neurocognitive performance accounts for 20–55% of the variance in functioning (Green et al., 2000; Nuechterlein et al., 2011), suggesting there could be individuals with schizophrenia who function well despite impaired neurocognition and that others may experience functional impairment despite intact neurocognitive performance. However, subgroups of schizophrenia defined jointly by neurocognitive ability and community functioning have never been examined.

The current study considered the convergence of premorbid neurocognition, current neurocognition, and functional impairment in a large sample of individuals with schizophrenia. This study aimed to, (1) examine demographic, symptom, and functional differences between neurocognitive subgroups based on both current and premorbid neurocognitive ability; (2) identify the proportion of individuals with schizophrenia whose functioning is better or worse than would be expected based on their neurocognitive ability; and (3) examine demographic and symptom differences between functional subgroups.

## 1 Methods

### 1.1 Participants

The current study aggregates participants from three previous NIMH-funded studies using equivalent measures: the Social Cognition Psychometric Evaluation study (SCOPE; Pinkham et al., 2016); the Validation of Everyday Real-world Outcomes study (VALERO; Harvey et al., 2011); and the EPI-Gen study conducted at the Johns Hopkins School of Medicine (Bowie et al., 2018). The SCOPE study assessed psychometric properties of social cognition tasks in schizophrenia, the VALERO study evaluated psychometric properties of various functional rating scales, and EPI-Gen examined genetic contributions to cognition and functioning in schizophrenia and bipolar disorder (only participants with schizophrenia were included in the current sample). Methods for each of these studies are described in more detail elsewhere (Bowie et al., 2018; Harvey et al., 2011; Pinkham et al., 2016; Pinkham et al., 2018).

The sample consisted of 410 participants from SCOPE, 195 from VALERO, and 423 from EPI-Gen; however, 74 participants were missing complete data, resulting in a final sample of 954 participants. All participants had a diagnosis of schizophrenia or schizoaffective disorder verified by a psychiatrist or clinical psychologist.

### 1.2 Measures

#### 1.2.1 Neurocognition

A subset of the MATRICS Consensus Cognitive Battery (Nuechterlein et al., 2008) assessed processing speed (Trail Making Test, Part A; Symbol Coding; and Category Fluency: Animal Naming), working memory (Letter-Number Span); and verbal learning (Hopkins Verbal Learning Test-Revised; HVLT-R). Verbal learning was assessed using the Rey Auditory Verbal Learning Test (Schmidt, 1996) in place of the HVLT-R in one sample (Bowie et al., 2018). These neurocognitive tests align with the three-domain structure of the MCCB – composed of processing speed, working memory, and learning (Lo et al., 2016; McCleery et al., 2015). All neurocognitive data were converted from raw scores to *z*-scores using published norms. Current neurocognitive functioning is represented by a composite *z*-score calculated from the average of the 5 neurocognitive tests. The WRAT-3 Reading *z*-score (Wilkinson, 1993) was used as an estimate of premorbid IQ.

#### 1.2.2 Community functioning

The Specific Level of Functioning scale (SLOF; Schneider and Struening, 1983) is a 43-item measure assessing domains of interpersonal relationships, participation in community and household activities, and work skills. The informant-rated scale of the SLOF was used. Each item is rated on a 5-point scale, with higher ratings indicating better functioning. The Total SLOF score was calculated as the mean of the three subscales: Interpersonal Relationships, Activities, and Work Skills. Then this total score was converted to a percentage of the total possible score for ease of interpretation.

#### 1.2.3 Psychiatric symptoms

The Positive and Negative Syndrome Scale (PANSS; Kay et al., 1987) is a 30-item assessor-rated measure assessing positive, negative, and general symptoms of psychosis. Items are rated on a scale from 1 to 7, with higher scores indicating greater psychopathology. The Beck Depression Inventory-II (BDI-II; Beck et al., 1996) was used as a measure of depressive symptoms. The BDI-II is a 21-item self-report questionnaire assessing common symptoms of depression. Items are rated on a scale from 0 to 3, with higher scores indicating greater severity of depression.

### 1.3 Data analysis

#### 1.3.1 Defining neurocognitive groups

Subgroups were created according to three combined criteria: current neurocognitive functioning, estimated premorbid IQ, and degree of deterioration in neurocognitive functioning compared to premorbid IQ. Current neurocognition was considered impaired if the neurocognitive composite *z*-score was more than 1 SD below the normative mean. Estimated premorbid IQ was considered impaired if the WRAT-3 Reading standard score was more than 1 SD below the normative mean. Deterioration was defined as a current neurocognitive functioning z-score greater than one standard error of measurement (SEM; Wilkinson, 1993) below the estimated premorbid IQ *z*-score. Following an approach similar to Weickert et al. (2000) and based on the above metrics, participants were classified into five groups: (1) Compromised – characterized by below average premorbid IQ, below average current neurocognitive functioning, and no deterioration from premorbid IQ; (2) Compromised + Deteriorated – characterized by below average premorbid IQ, below average current neurocognitive functioning, and deterioration; (3) Preserved – characterized by average premorbid IQ, average neurocognitive functioning, and no deterioration; (4) Preserved + Deteriorated – characterized by average premorbid IQ, average neurocognitive functioning, and deterioration; and (5) Deteriorated – characterized by average premorbid IQ, impaired neurocognitive functioning, and deterioration.

#### 1.3.2 Defining functional groups

Cluster analyses were performed to examine whether participants could be classified into discrete groups based on their neurocognitive test performance and community functioning. As this type of grouping has not been examined before, the data-driven cluster approach was used to classify subgroups. The SLOF Total score was transformed to a *z*-score based on the mean of the sample. Outliers were removed if they were more than 3 SDs below the mean (*N* = 23). First, a hierarchical cluster method was applied to help determine the number of clusters. Ward's method using squared Euclidean distance was applied to the neurocognitive composite *z-*score and SLOF Total *z*-score to minimize the total within-cluster variance (Aldenderfer and Blashfield, 1984). Following this, k-means clustering was used as the primary technique to determine the stability of the final solution.

Discriminant analysis was used to confirm the cluster results and validate the classification accuracy (Sauvé et al., 2018; Uren et al., 2017). The discriminant analysis model was built using cluster grouping as the dependent variable and neurocognition and community functioning variables as independent variables. The SLOF Total Percentage variable was non-normally distributed as identified by inspection of the Shapiro-Wilk test and Q-Q Plots and was transformed for normality using Templeton's (2011) two-step approach. Step 1 of this approach involves transforming the SLOF Total Percentage score into a percentile rank to create uniformly distributed probabilities. Then, an inverse-normal transformation was applied to the results of Step 1 to form a variable consisting of normally distributed z-scores.

#### 1.3.3 Group comparisons

As the groups varied widely in size, Kruskal-Wallis tests were used to compare the neurocognitive groups on demographic information, neurocognitive functioning, real-world functioning, and symptoms. Pairwise comparisons were used for post-hoc analysis. One-way analysis of variance (ANOVAs) was used to compare the functional clusters on demographic information and symptoms. Tukey's test was used for post-hoc analysis. Chi-square analyses were used to test differences between the groups for categorical variables. Significance values were adjusted by the Bonferroni correction for multiple tests.

## 2 Results

### 2.1 Neurocognitive groups

Comparisons between groups are presented in Table 1. The number of participants that met criteria for each group was: Compromised (*N* = 85); Compromised + Deteriorated (*N* = 83); Preserved (*N* = 66); Preserved + Deteriorated (*N* = 261); and Deteriorated (*N* = 459).

**Table 1:** Demographics, neurocognition, functioning, and symptoms across neurocognitive group.

	Compromised (*N* = 85, 8.9%)	Compromised + Deteriorated (*N* = 83, 8.7%)	Preserved (*N* = 66, 6.4%)	Preserved + Deteriorated (*N* = 261, 27.4%)	Deteriorated (*N* = 459, 48.1%)	H	Post-hoc (pairwise)
	M (SD)	M (SD)	M (SD)	M (SD)	M (SD)		
Age	43.61 (10.98)	46.86 (10.05)	41.76 (10.59)	45.30 (12.53)	47.22 (11.72)	20.09⁎⁎	3 < 5
Sex							
Male N (%)	50 (59%)	56 (67%)	39 (59%)	170 (65%)	316 (69%)	Chi-square = 5.18	n.s.
Female N (%)	35 (41%)	27 (33%)	27 (41%)	91 (35%)	143 (31%)		
Years of education	12.41 (1.89)	11.77 (2.01)	13.36 (2.44)	14.74 (2.42)	13.62 (2.48)	129.21⁎⁎⁎	1,2,3,5 < 4
							1,2 < 5
							2 < 3
WRAT-3 Reading SS	74.65 (7.69)	79.57 (5.66)	96.82 (8.25)	108.29 (6.77)	101.81 (8.68)	504.67⁎⁎⁎	1 < 3,4,5
							2 < 3,4,5
							3 < 4,5
							5 < 4
Neurocog composite *z*-score	−1.42 (0.33)	−2.29 (0.47)	−0.24 (0.43)	−0.47 (0.39)	−1.65 (0.53)	676.23⁎⁎⁎	2 < 1,3,4,5
							5 < 3,4
							1 < 3,4
SLOF Total %	76.37 (12.41)	75.61 (12.41)	82.49 (12.19)	85.05 (10.19)	80.42 (12.51)	42.56⁎⁎⁎	1,2,5 < 4
PANSS Positive	16.61 (5.85)	17.25 (5.77)	15.44 (5.77)	14.31 (5.32)	15.99 (5.65)	24.16⁎⁎⁎	4 < 1,2,5
PANSS Negative	15.20 (6.05)	19.41 (7.49)	12.77 (4.83)	12.88 (5.03)	15.60 (5.93)	85.86⁎⁎⁎	1,3,4,5 < 2
							4 < 1,5
							3 < 5
PANSS General	30.81 (8.08)	33.44 (7.53)	31.72 (8.66)	28.52 (7.03)	30.83 (7.82)	31.84⁎⁎⁎	4 < 2,5
PANSS Total	62.62 (15.99)	69.77 (15.33)	59.69 (15.20)	55.61 (13.35)	62.29 (14.94)	63.50⁎⁎⁎	1,4,5 < 2
							4 < 1, 5
BDI-II Total	14.85 (12.07)	16.03 (12.47)	15.83 (13.20)	11.14 (9.19)	14.25 (11.21)	16.22⁎⁎	4 < 2,5

Note. WRAT-3 Reading SS = Scaled Score; SLOF = Specific Level of Functioning Scale; PANSS = Positive and Negative Syndrome Scale; BDI-II = Beck Depression Inventory; 1 = Compromised; 2 = Compromised + Deteriorated; 3 = Preserved; 4 = Preserved + Deteriorated; 5 = Deteriorated.

⁎⁎ *p* < .01.

⁎⁎⁎ *p* < .001.

### 2.2 Demographic, symptom, and functional comparisons between neurocognitive groups

The Preserved group was significantly younger than the Deteriorated group. The Preserved + Deteriorated group had significantly more years of education compared to every other group. The Preserved group had significantly more years of education than the Compromised + Deteriorated group and the Deteriorated group had significantly more years of education compared to both Compromised groups. There were no significant sex differences between groups.

The Compromised + Deteriorated group had the fewest years of education, lowest community functioning score, and most severe symptoms of the entire sample. The Compromised group also had fewer years of education, lower community functioning, and more severe symptoms on PANSS Positive and PANSS Total compared to the Preserved, Preserved + Deteriorated, and Deteriorated groups.

Both Preserved and Preserved + Deteriorated groups had better community functioning and less severe symptoms on the PANSS Positive, Negative, and Total scales compared to the Compromised groups. The Preserved + Deteriorated group had significantly lower scores than both Compromised groups on the PANSS Positive, Negative, Total, and BDI-II Total Scores. The Preserved + Deteriorated group additionally had lower PANSS General scores than the Compromised + Deteriorated group. The Deteriorated group did not significantly differ from either of the Compromised groups.

### 2.3 Functional groups cluster analysis

**Fig. 1:**
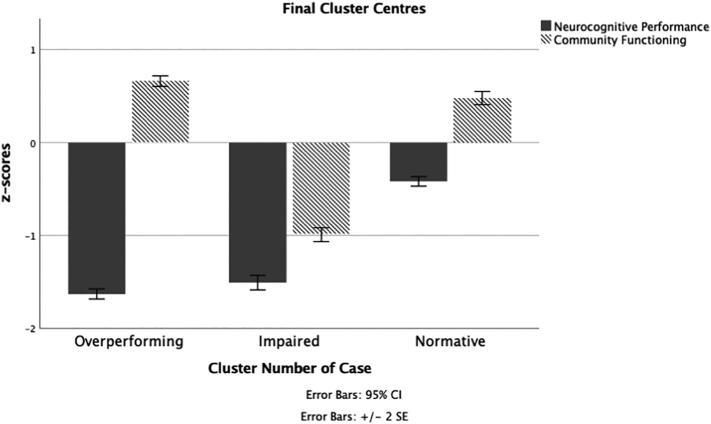
Neurocognitive and community functioning scores by cluster.

A hierarchical cluster analysis using squared Euclidean distances resulted in a dendrogram suggesting 2, 3, and 4-cluster solutions. Then, a series of k-means cluster analyses specifying 2, 3, and 4 clusters were performed. To identify an optimal number of clusters from these choices, we visually inspected scatterplots, compared Silhouette indices (Rousseeuw, 1987), and considered clinical interpretability. A 3-cluster solution maximized separation between cluster centres (Fig. 1).

Cluster 1 (*N* = 294) had higher community functioning (SLOF Total Percentage = 89.11%) and impaired neurocognition (neurocognitive composite *z*-score = −1.64), representing participants who are overperforming what would be expected based on neurocognitive ability. Cluster 2 (*N* = 280) had both impaired community functioning (SLOF Total Percentage = 66.63%) and neurocognition (neurocognitive composite *z*-score = −1.50) representing participants who are both functionally and cognitively impaired. Cluster 3 (*N* = 284) had both higher community functioning (SLOF Total Percentage = 87.02%) and intact neurocognition (neurocognitive composite *z*-score = −0.39) representing participants who are higher functioning and cognitively intact.

### 2.4 Validation

**Fig. 2:**
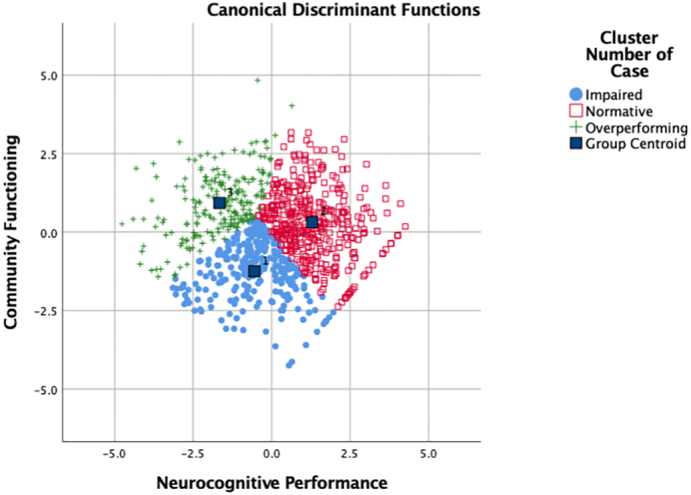
Discriminant plot of K-means 3 cluster solution.

The discriminant function plot of the final 3-cluster solution indicated cohesive clusters with a concentration of cases around each centroid and yielded a correct classification rate of 97.3% (Wilks' Lambda = 0.21, *p* < .000; Fig. 2).

### 2.5 Symptom and demographic factors associated with functional clusters

Clusters were compared on demographic variables and psychiatric symptomatology (Table 2). The Normative group had the most years of education, followed by the Overperforming group, while the Impaired subgroup had the fewest. Both the Overperforming and Normative groups had lower PANSS Positive, General, Total, and BDI-II scores compared to the Impaired group. The Overperforming group had a significantly more severe PANSS Negative score than the Normative group.

**Table 2:** Comparison of demographics and symptoms by functionally determined cluster.

	Overperforming (*N* = 296, 34.3%)	Impaired (*N* = 279, 32.3%)	Normative (*N* = 289, 33.4%)	F	Post-hoc (Tukey's)
	M (SD)	M (SD)	M (SD)		
Age	46.44 (11.52)	45.57 (11.31)	44.37 (12.06)	2.30	n.s.
Sex					
Male N (%)	207 (71%)	177 (63%)	179 (63%)	Chi-square = 4.49	n.s.
Female N (%)	87 (29%)	103 (37%)	105 (37%)		
Years of education	13.69 (2.50)	12.84 (2.10)	14.34 (2.55)	27.16⁎⁎⁎	Im < O < N
PANSS Positive	15.18 (5.41)	16.93 (5.50)	14.64 (5.57)	13.19⁎⁎⁎	O, N < Im
PANSS Negative	14.44 (5.16)	16.73 (6.49)	12.88 (5.15)	33.11⁎⁎⁎	N < O < Im
PANSS General	28.79 (7.05)	32.87 (7.59)	29.32 (7.43)	25.44⁎⁎⁎	O, N < Im
PANSS Total	58.40 (13.33)	66.38 (14.48)	56.75 (13.77)	38.24⁎⁎⁎	O, N < Im
BDI-II Total	12.20 (10.22)	17.63 (12.24)	12.06 (9.89)	23.97⁎⁎⁎	O, N < Im

Note. n.s. = not significant; PANSS = Positive and Negative Syndrome Scale; BDI-II = Beck Depression Inventory; O = Overperforming; N = Normative; Im = Impaired.

⁎⁎⁎ p < .001.

### 2.6 Association between neurocognitive and functional clusters

We investigated neurocognitive group membership within each functional cluster. Individuals in the Normative functional cluster mostly belonged to the Preserved subgroups. The Overperforming functional cluster appeared to be mostly composed of the Deteriorated subgroup, who had good premorbid IQ but current impairment. The Impaired functional cluster was composed predominantly of the Compromised and Deteriorated neurocognitive subgroups (Table 3).

**Table 3:** Group membership across neurocognitive groups and functional clusters.

	Overperforming (*N* = 296)	Impaired (*N* = 269)	Normative (*N* = 257)
Compromised	30 (10%)	38 (14%)	3 (1%)
Compromised & Deteriorated	30 (10%)	34 (13%)	0 (0%)
Preserved	1 (0.5%)	11 (4%)	45 (18%)
Preserved & Deteriorated	4 (1.5%)	29 (11%)	201 (78%)
Deteriorated	231 (78%)	157 (58%)	8 (3%)

Note. Total *N* = 822.

## 3 Discussion

The sample was neurocognitively heterogeneous, with 65% displaying overall neurocognitive impairment and 84% experiencing impairment relative to premorbid levels. Only 6.4% of the sample displayed no indication of current cognitive impairment or decline from premorbid levels. The two Compromised subgroups had poorer community functioning, less education, and greater severity of psychotic symptoms compared to the Preserved groups. The Deteriorated group was intermediate between the Compromised and Preserved subgroups across all measures except depression and general psychopathology.

Incorporating neurocognitive functioning with community functioning in a cluster analysis resulted in three subgroups, which included: (1) an Overperforming cluster, with impaired neurocognition but higher community functioning; (2) a Normative cluster, with intact neurocognition and higher community functioning; and (3) an Impaired cluster, with impaired neurocognition and community functioning. Approximately 30% of the sample was found to be functionally overperforming what would be expected based on their neurocognitive performance. Importantly, both the Overperforming and Normative groups had comparable SLOF scores to a sample of HCs (Ludwig et al., 2017). In this study, HCs had a total percentage score equivalent to 92% with a standard deviation of approximately 5%. Thus, the Overperforming and Normative scores are within one standard deviation of the HC scores and could be interpreted as lying within normal limits. Individuals with better community functioning, regardless of neurocognitive ability, had less severe symptoms compared to those with functional impairments. The Overperforming group had less severe negative symptoms than the Normative group but did not differ on any other symptom measures.

Most of the sample had premorbid neurocognitive performance falling in the average range, which is consistent with meta-analytic evidence of premorbid IQ in schizophrenia (Woodberry et al., 2008; Harvey et al., 2006a). Integrating those data with the established neurocognitive impairments (Fioravanti et al., 2012; Reichenberg, 2010; Schaefer et al., 2013) indicates that some degree of decline associated with the development of a schizophrenia-spectrum disorder is typical. In the present study approximately 50% of the sample performed below 1.5 SDs below the mean on neurocognition but had a WRAT reading score in the normative range. Overall, regardless of current or premorbid neurocognitive ability, 84.2% of the sample was classified as Deteriorated and only 6.4% of the sample was cognitively preserved with no evidence of deterioration. Even among this Preserved subgroup, 41% displayed impairment of at least 1.5 SD on at least one cognitive subtest, indicating that even though they are not globally impaired, there appears to be some selective impairment in specific domains.

Important differences across several demographic, clinical, and functional variables were observed among neurocognitive subgroups. The Compromised subgroup had the highest level of negative symptoms and poorest community functioning of the entire sample, which is unsurprising given the strong link between neurocognition, negative symptoms, and functional outcomes (Harvey et al., 2006b; Lin et al., 2013; Ventura et al., 2009). The Compromised subgroups also had the fewest years of education of the groupings, which would be expected based on their low WRAT scores. In contrast, the Preserved subgroups had the lowest level of symptoms across all PANSS subscales, the best community functioning, and most years of education, consistent with studies showing that individuals with preserved neurocognitive functioning tend to have higher educational attainment (Leeson et al., 2011; Weickert et al., 2000) and superior clinical and functional outcomes (Gilbert et al., 2014). This replicates observations from prior research outlining socio-clinical factors associated with various cognitive subgroups, the most consistent of which being years of education (Hoti et al., 2004; Reser et al., 2015), community functioning (Gilbert et al., 2014; Uren et al., 2017), and negative symptoms (Potter and Nestor, 2010; Reser et al., 2015; Uren et al., 2017). In all cases, the higher-functioning cognitive subgroups had greater educational attainment, better community functioning, and lower severity of negative symptoms compared to the cognitively impaired subgroups. The same clinical differences are consistently observed in first-episode psychosis samples, as well (Reser et al., 2015; Uren et al., 2017), indicating that they are not a product of illness chronicity.

To our knowledge, this is the first study to perform a cluster analysis incorporating both neurocognitive and functional variables in schizophrenia. The results demonstrated that there are a significant number of individuals with schizophrenia who are functionally overperforming what would be expected based on their neurocognitive abilities. These individuals were more likely to have had average or above average range estimated premorbid IQ. Importantly, those who had higher community functioning alongside impaired neurocognitive functioning did not significantly differ from those with intact neurocognitive functioning across any symptom domains other than PANSS Negative subscale. The fact that negative symptoms were the only variable which separated each of the three clusters highlights the link between negative symptoms, neurocognitive functioning, and community functioning (Harvey et al., 2006b). However, these results suggest that negative symptoms may be more closely linked with neurocognition than functioning, as the Overperforming group presented with intermediate levels of negative symptoms but were still functioning better in the community relative to other individuals with schizophrenia spectrum illness. Importantly, it seems that intact neurocognitive functioning is not a prerequisite for intact community functioning.

The finding that 34% of this sample had higher community functioning despite significant neurocognitive impairment is important to understand the heterogeneity of schizophrenia. It is unclear how this group is able to function relatively well despite impaired neurocognition performance, and future research could examine what factors contribute to this group's higher functioning. One possible contribution is a higher premorbid IQ, as suggested by the association with the deteriorated neurocognitive group, or superior functioning in relevant neurocognitive domains, such as executive functioning. The cognitive battery was limited by those measures included in all studies and executive functioning was not assessed in the current sample. It is possible that the Overperforming group was characterized by better executive functioning skills as these are known to have a very strong association with functional outcome (Kluwe-Schiavon et al., 2013). Another possibility is age at the first episode of psychosis since older age of onset may provide more opportunities for individuals to develop functional skills before impairments develop, however, this data was not available for the current sample. Possible psychosocial factors may include extra support from caregivers and good social networks (Vázquez Morejón et al., 2018), as well as higher levels of self-esteem, self-efficacy, and resilience (Vracotas et al., 2012). These findings also highlight the need to investigate emerging factors deemed to be important determinants of functional outcomes, such as social cognition (Halverson et al., 2019) or introspective accuracy (Silberstein and Harvey, 2019).

Factoring individual differences into treatment selection has potential utility in prediction of treatment response for cognitive interventions. Up to 25% of treated individuals do not respond to treatments such as cognitive remediation (CR; Wykes et al., 2011) and tailoring the CR approach to one's current level of cognitive functioning may improve outcomes. There is evidence to suggest that subgroups with lower baseline neurocognition demonstrate greater improvements in cognitive performance after a course of CR (Pillet et al., 2015; Twamley et al., 2011; DeTore et al., 2019). Additionally, it is possible that certain modalities of CR (e.g., self-guided training versus strategy monitoring; paired with psychotherapy or not) are better suited for different subgroups based on the presence of different cognitive, functional, and symptom presentations (Best and Bowie, 2017; Medalia et al., 2018). Such subgroups may also explain differential response to types of CR targeting higher- or lower-order cognitive domains (Best et al., 2019) and could be used to individually tailor the treatment approach. Moreover, as cognitive impairments appear to be more responsive to remediation during early stages of psychosis rather than in later periods of chronicity, there is potential utility in using such individual differences to guide early intervention efforts (Wykes et al., 2018). This could improve remediation success during this critical period by providing targeted CR to those who would benefit most.

The current study should be interpreted with consideration of several limitations. The study only included outpatients, which may have resulted in a higher functioning sample than if inpatients had been included. Premorbid neurocognition was only estimated using the WRAT and was not assessed prior to illness onset. This study did not include any measures of social cognition or executive functioning and only one measure of functioning. Additionally, since this combined sample did not include a healthy control group, we could not know the absolute level of functioning and whether it was within normative ranges. Previous studies of performance-based functioning have found that some individuals with schizophrenia overlap with healthy controls in the normal range (Miller et al., 2021; Vella et al., 2017), and future studies could examine the observed clusters in healthy controls. Future research could also examine the subgroups longitudinally to investigate the temporal stability of these groupings. The assessment measures were limited due to combining multiple datasets, however, this also allowed for a large enough sample to examine subgroups.

## 4 Conclusions

While impairments in neurocognitive and community functioning are common in schizophrenia, the distribution and degree of impairment across these domains is highly heterogeneous, and many individuals demonstrate normal-range and/or relatively better functioning. Across the neurocognitive subgroups, 66.3% demonstrated current neurocognitive impairment. When community functioning was added to the classification procedure, we found that 34% of the sample was functionally overperforming what would be expected based on their neurocognitive ability. These findings demonstrate that there are multiple ways of defining impairment and provide support for differentiating between clinical groups based on criteria such as neurocognitive and community functioning. Understanding these individual differences and how they can moderate treatment outcome has important implications for assessment and cognitive interventions.

## CRediT authorship contribution statement

All authors contributed to and approved the final manuscript. All authors contributed to the conceptualization of the study. SR wrote the first draft the manuscript. CAD, TLP, DLP, AEP, and PDH conceptualized and conducted the original studies that the current manuscript is based on. All authors contributed to the writing of the final manuscript.

## Funding sources

The SCOPE study was funded by the 10.13039/100000025National Institute of Mental Health, Grant/Award Number: R01MH093432. The VALERO study was supported by the 10.13039/100000025National Institute of Mental Health, Grant/Award Numbers: MH078775 and MH078737. The EPI-Gen study was supported by the 10.13039/100000025National Institute of Mental Health, Grant/Award Number: R01MH079784.

## Declaration of competing interest

Sylvia Romanowska – No conflicts to report.

Michael W. Best – No conflicts to report.

Christopher R. Bowie – In the past 5 years, Dr. Bowie has received consulting fees from Pfizer, Boehringer Ingelheim, and Lundbeck. He has received grant support from Pfizer, Lundbeck, and Takeda and research support from SBT Pro in kind research licenses. He has received book royalties from the Oxford University Press.

Colin A. Depp – No conflicts to report.

Thomas L. Patterson – No conflicts to report.

David L. Penn – No conflicts to report.

Amy E. Pinkham – No conflicts to report.

Philip D. Harvey – Dr. Harvey has received consulting fees or travel reimbursements from Alkermes, Bio Excel, Boehringer Ingelheim, Karuna Pharma, Merck Pharma, Minerva Pharma, SK Pharma, and Sunovion (DSP) Pharma in the past year. He receives royalties from the Brief Assessment of Cognition in Schizophrenia (Owned by WCG Verasci, Inc. and contained in the MCCB). He is chief scientific officer of i-Function, Inc.
